# *Plasmodium vivax* gametocytes in the bone marrow of an acute malaria patient and changes in the erythroid miRNA profile

**DOI:** 10.1371/journal.pntd.0005365

**Published:** 2017-04-06

**Authors:** Barbara Baro, Katrien Deroost, Tainá Raiol, Marcelo Brito, Anne C. G. Almeida, Armando de Menezes-Neto, Erick F. G. Figueiredo, Aline Alencar, Rodrigo Leitão, Fernando Val, Wuelton Monteiro, Anna Oliveira, Maria del Pilar Armengol, Carmen Fernández-Becerra, Marcus V. Lacerda, Hernando A. del Portillo

**Affiliations:** 1 Fundação de Medicina Tropical Dr. Heitor Vieira Dourado (FMT-HVD), Manaus, Amazonas, Brazil; 2 Fundação Oswaldo Cruz - Instituto Leônidas e Maria Deane (FIOCRUZ-Amazonas), Manaus, Amazonas, Brazil; 3 Universidade do Estado do Amazonas (UEA), Manaus, Amazonas, Brazil; 4 ISGlobal, Barcelona Ctr. Int. Health Res. (CRESIB), Hospital Clínic - Universitat de Barcelona, Barcelona, Spain; 5 Fundação Hospitalar de Hematologia e Hematoterapia do Amazonas (HEMOAM), Manaus, Amazonas, Brazil; 6 Institut d’Investigació Germans Trias i Pujol (IGTP), Badalona, Spain; 7 ICREA, Barcelona, Spain; Walter and Eliza Hall Institute, AUSTRALIA

*Plasmodium vivax* is the most widely distributed human malaria parasite and responsible for large amounts of disease and burden [1]. The presence of *P*. *vivax* in the bone marrow was first noticed in the late 19th century [2], and examinations of sternal bone marrow aspirates were performed as an accessory to examinations of peripheral blood in malaria, including *P*. *vivax* [3]. Since then, little progress has been made in studying *P*. *vivax* infections in this tissue. One report explored accumulation of dyserythropoietic cells in anaemic infected patients [4]. In addition, two case studies reported *P*. *vivax* infections after autologous bone marrow transplantation [5][6], and a third one documented an accidental *P*. *vivax* infection due to bone marrow transplantation between a malaria-infected donor and a malaria-free receptor [7]. In Brazil, one patient with persistent thrombocytopaenia and an enlarged spleen was diagnosed with chronic *P*. *vivax* malaria after the finding of schizonts in the bone marrow aspirate [8]. In all these reports and case studies, however, parasite loads and life stages found in the bone marrow were not investigated, and no molecular tools were available to rule out mixed infections or to characterize specific parasite stages.

## Description of case

To gain insight into *P*. *vivax* infections in the bone marrow, we performed a morphological and molecular study of bone marrow aspirates taken from a 46-year-old man who was diagnosed with *P*. *vivax* (13,280 parasites/μL) at the tertiary hospital of Fundaçao de Medicina Tropical Dr. Heitor Vieira Dourado (FMT-HVD), Manaus, Amazonas, Brazil. Bone marrow aspirate (4 mL) and peripheral blood (15 mL) were collected before treatment was administered following the national guidelines (1,500 mg of choloroquine over 3 days, 30 mg of primaquine per day for 7 days). At convalescence, 42 days after treatment, bone marrow aspirate and peripheral blood samples were obtained for comparison from this same individual. Parasitaemia at day 42 was negative on microscopy and quantitative polymerase chain reaction. Relevant haematological and biochemical parameters are described in S1 Table.

## Ethics statement

This patient was enrolled in a larger study designed to understand determinants of anaemia in acute *P*. *vivax* infection. The study was approved by the Institutional Reviewing Board of the Fundação de Medicina Tropical Dr. Heitor Vieira Dourado (FMT-HVD), Manaus, Amazonas, Brazil and the National Committee of Ethics in Science and Technology (CONEP Process No.: 25.001.011.792/2009-15). The patient was fully informed on the aims of the study and signed an informed consent agreement after understanding the risks of the procedure. The patient also consented to his case being published.

## Presence of *P*. *vivax* parasites in the bone marrow during active infection

**Fig 1:**
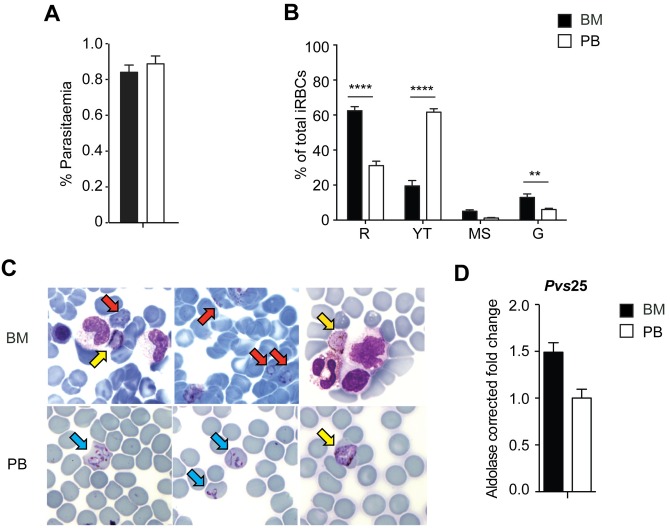
Comparison of *P*. *vivax* load and life stages in bone marrow aspirate and peripheral blood on admission. **A**. Parasitaemia in bone marrow aspirate and peripheral blood at the day of admission. **B**. Parasite stage distribution in bone marrow and peripheral blood. n = 800 iRBCs. R = rings, YT = young trophozoites, MS = mature trophozoites and schizonts, G = gametocytes. **C**. Representative Giemsa-stained images of *P*. *vivax* in the bone marrow (BM, upper row) illustrating rings (red arrows) and gametocytes (yellow arrows) and in peripheral blood (PB, lower row) illustrating young trophozoites (blue arrows) and gametocytes (yellow arrow). Arrows indicate infected cells. **D**. Relative RT-qPCR quantification of *pvs25* transcripts in bone marrow and peripheral blood samples obtained at admission. *pvs25* transcript levels were normalized by amplifying aldolase; bone marrow quantification was expressed as fold change function of peripheral blood quantification. Calculated bone marrow aspirate purity was 80%. BM purity = [1 - (erythrocyte-BM/erythrocytes-PB) x (leukocytes-PB/leukocytes-BM)] x 100. Statistical tests were performed with GraphPad Prism software. Paired t-tests were used to compare differences between two groups, whereas two-way ANOVA with Sidak test for correction for multiple comparisons was used in case of more then two groups. Data in graphs are shown as mean ± standard error of the mean. *p* < 0.05 was regarded as statistically different. **: *p* < 0.01 and ****: *p* < 0.0001.

To avoid confounding, we first excluded coinfection with *P*. *falciparum* by standard qPCR of 18S rDNA [9]. Next, we determined parasitaemia in peripheral blood and bone marrow by counting 15,000 enucleated red blood cells (RBCs) (i.e., reticulocytes and erythrocytes) in Giemsa-stained thin blood smears. The enucleated RBC content was similar between bone marrow (4.08 x 10^6^/uL) and peripheral blood (4.6 x 10^6^/uL) samples as determined by hemocytometry, and no invasion was observed in nucleated erythroid precursor cells. Three different people independently counted 5,000 RBCs each. Parasitaemia was <1% and similar in bone marrow and peripheral blood (Fig 1A); yet, clear differences in parasite stage distribution in each compartment were observed. To investigate the proportion of the different stages in more detail, stage differentiation was performed by counting 800 infected RBCs. Rings (*p* < 0.0001) and gametocytes (*p* < 0.01) were significantly more abundant in the bone marrow aspirate as compared to peripheral blood (given these compartments had similar parasitaemia), whereas young trophozoites were predominantly present in peripheral blood (*p* < 0.0001) (Fig 1B). Representative images of these stages in the bone marrow and peripheral blood are shown in Fig 1C. It is worth mentioning that multiple-infected cells containing up to five parasites were also observed in peripheral blood and bone marrow (S1A and S1B Fig). The enrichment of ring stages in the bone marrow aspirate is in agreement with studies of reticulocyte-prone malaria parasites, including *P*. *vivax*, demonstrating preferential invasion of parasites in reticulocytes expressing high levels of Cluster of Differentiation 71 that are mostly found in the bone marrow [10][11].

As gametocytes of *P*. *vivax* can be morphologically confused as schizonts, the gametocyte fraction in each compartment was further quantified by RT-qPCR using primers amplifying the gametocyte specific transcript *pvs25* [12]. To normalize for parasite content, *pvs25* transcript levels were quantified relative to the levels of the housekeeping gene coding for aldolase, expressed similarly throughout all asexual blood stages [13]. Bone marrow quantification was expressed as fold change function of peripheral blood quantification. As shown in Fig 1D, *pvs25* D0 transcripts were enriched in bone marrow as compared to peripheral blood, showing higher accumulation of transmission stages in this milieu. *Pvs25* RT-qPCR quantification on D42 was not reliable (Ct values >35, unspecific amplification) and thus was considered negative.

In *P*. *falciparum*, early studies have shown enrichment of immature gametocytes in the bone marrow of infected children [14], and this observation has been confirmed by molecular tools [15][16]. Of interest, gametocytes’ immature forms were enriched in the bone marrow, whereas the mature gametocyte’s stage V was commonly found in peripheral blood. The results from our studies show that *P*. *vivax* gametocyte stage-infected cells are also enriched in the bone marrow as compared to peripheral blood during the acute infection of this patient. Presently, however, there are no reliable morphological or molecular markers to distinguish between maturation stages of *P*. *vivax* gametocytes. Regardless, it is largely assumed that *P*. *vivax* gametocytes in the peripheral blood parallel the beginning of malaria-associated symptoms, leading to an evolutionary benefit of this species regarding early vector transmission before treatment. Considering this rationale, it was not generally assumed that *P*. *vivax* gametocytes could accumulate in the bone marrow. Our data thus suggest that the bone marrow could also be a reservoir for gametocytes during *P*. *vivax* infections. Whether it can be a niche for gametocyte production and/or maturation and whether gametocytes can actually sequester in the bone marrow will be very interesting to investigate in future experiments.

## Erythrocyte precursors changes during the active infection

Morphological analysis of bone marrow aspirates from human patients presenting anaemia has shown that infection with *P*. *vivax* induces dyserythropiesis and ineffective erythropoiesis [4]. Before drug treatment, an increase in dyserythropietic cells (5%) was observed by microscopic examination of the bone marrow aspirate during infection. Cell changes included the presence of erythroblasts with binucleated or budding nuclei and cytoplasmic bridges between erythroblasts (S1D Fig). Furthermore, erythroblast differentiation stages were counted to examine whether inefficient erythropoiesis was present (n = 1,000 erythroblasts). Proerythroblasts, basophilic erythroblasts, polychromatic erythroblasts, and orthochromatic erythroblasts were 7.1%, 14.8%, 38.5%, and 39.6%, respectively, and did not follow the expected 1:2:4:8 ratio, indicating a problem at the level of polychromatic and orthochromatic erythroblasts. These results thus suggest inefficient erythropoiesis during an active infection, as has been reported previously for *P*. *vivax* [4]. Noticeably, the haemoglobin (Hb) level of this patient at the day of admission was 13.98 g/dL as opposed to 15.86 g/dL at convalescence 42 days later. Of note, after drug treatment, Hb levels dropped to 12.04 g/dL. Thus, even though this patient was not clinically anaemic at the time of recruitment, it is clear that during infection he had a drop of his normal Hb level, potentially explaining the dyserythropoiesis we observed.

## Infection in the bone marrow is associated with transcriptional changes

**Fig 2:**
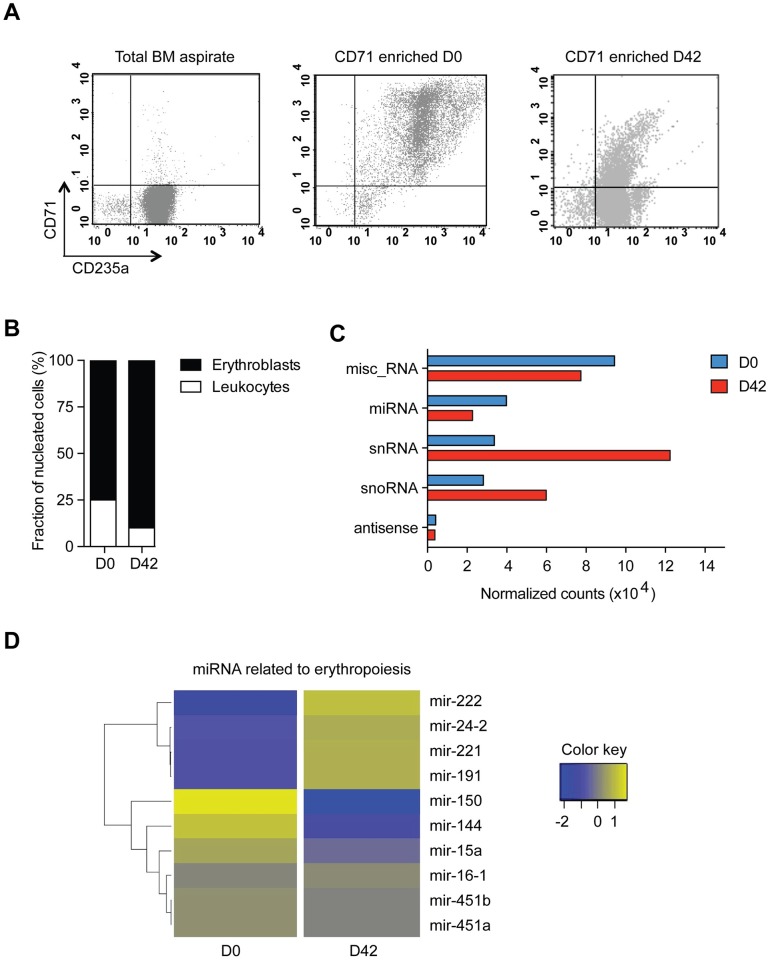
Small RNA profile of bone marrow CD71+ erythroid precursor cells on admission and at convalescence. **A**. Flow cytometry plots demonstrating the enrichment of erythroid cells as stained with CD235a/Glycophorin A-FITC and CD71-PE showing the initial bone marrow sample at D0 and the CD71+ enriched fraction after purification of CD71-coated beads for D0 and D42 samples. **B**. Fraction of leukocyte contamination in the CD71-enriched fraction for D0 and D42 as determined by counting n = 500 nucleated cells by microscopy on Giemsa-stained slides. **C**. Normalized read counts of small RNA categories present at D0 and D42. misc_RNA: miscellaneous other RNA, miRNA: microRNA, snRNA: small nuclear RNA, snoRNA: small nucleolar RNA, antisense: antisense RNA. **D**. Heatmap of erythropoiesis-related miRNA normalized counts were generated using the package gplots in R. Fold changes were calculated as the normalized read counts of D42/D0 ratio on a logarithmic scale for each miRNA.

Increasing evidence on the role of micro (mi)RNAs in controlling erythropoiesis is now available [17][18]. In order to address whether bone marrow transcriptional changes related to erythropoiesis during infection were present in this patient, the expression profiles of small RNAs during the acute attack and at convalescence were determined. To avoid confounding we first purified erythroid precursor cells from the bone marrow aspirates through affinity chromatography with magnetic CD71-labelled beads. We obtained a cell suspension containing more than 90% erythroid cells, including all erythrocyte precursors from proerythroblasts to reticulocytes, as observed by flow cytometry analysis and microscopy. Mature erythroblasts and reticulocytes were the most abundant cell populations (Fig 2A and 2B). Following purification, RNA was extracted and small RNAs were sequenced using the MiSeq platform. The complete bioinformatics pipeline and tools for analyses are shown in S2 Fig.

In summary, out of the 2,191,943 clipped reads from day 0 and 2,988,148 clipped reads from day 42, 721,739 (32.93%) and 1,090,129 (36.48%) could be uniquely mapped to the human reference genome, respectively. Relative gene expression was calculated by dividing the read count for each gene, considering all non-coding (nc)RNA genes with at least three reads mapped within their location, by the raw read count ratio of D0 to D42 libraries. Interestingly, a very distinct profile of small RNAs was observed under infection compared to convalescence: miRNAs and miscellaneous (misc)RNAs were induced during infection, while small nuclear (sn)RNAs and small nucleolar (sno)RNAs were halted (Fig 2C). Analysis of miRNAs related to erythropoiesis revealed a distinct series of differentially expressed miRNAs during *P*. *vivax* infection in this patient (Fig 2D). Mir-221/222, mir-24, and mir-191, which are normally downregulated during erythroid maturation, were decreased during *P*. *vivax* infection compared to convalescence. In contrast, mir-144, which is upregulated during erythropoiesis, was found to be increased. Mir-150, which drives megakaryocyte formation while inhibiting erythropoiesis, was found to be increased as well during infection. Hence, our results indicate an altered miRNA profile regarding bone marrow erythropoiesis during the acute *P*. *vivax* infection in this patient.

## Conclusion

Human studies of *P*. *vivax* in the bone marrow are scarce, even though its presence in this tissue was first noticed more than a century ago. Here, we describe a morphological and molecular study of bone marrow aspirates from a *P*. *vivax* patient with an unusually high parasitemia. Samples were taken on the day of admission, before drug treatment (13,280 parasites/μL), and 42 days after drug treatment, after clearance of parasitemia. On admission, parasitemia was similar in peripheral blood and bone marrow; yet, ring- and schizont-infected cells, as well as gametocytes, were significantly more abundant in the bone marrow. Morphological analysis revealed signs of inefficient erythropoiesis and dyserythropoiesis. In addition, transcriptional analysis of RNA extracted from marrow CD71+ cells revealed significant changes in miRNA and small RNA profiles on admission and convalescence. All together, these data show the presence of *P*. *vivax* in the marrow of this patient, providing the first quantification of parasite stages found in this tissue and demonstrating that its presence influences transcriptional changes of miRNAs involved in erythropoiesis.

**Key learning points**

- *Plasmodium vivax* parasites can be found in the bone marrow during an active infection.
- As with *P*. *falciparum*, the bone marrow could be a niche for gametocyte production and maturation and/or a reservoir in *P*. *vivax* infections.
- Molecular tools for specific quantification of different gametocyte stages are needed to address whether gametocyte-immature forms of *P*. *vivax* are enriched in the bone marrow similar to *P*. *falciparum*.
- The presence of *P*. *vivax* in the bone marrow of this patient was associated with transcriptional changes of miRNAs involved in erythropoiesis.

## Supporting information

S1 Table Haematological and biochemical parameters from peripheral blood on admission and at convalescence. (PDF)

S1 Fig Multiple ring-infected cells and signs of dyserythropoiesis. **A**. Percentages of single vs multiple ring-infected cells in the bone marrow and peripheral blood. **B**. Numbers of rings in individual infected cells in bone marrow and peripheral blood. Only infected cells containing multiple ring stages were used for confident counting (n = 500 iRBCs), although multi-invasion was observed in all parasite stages. **C**. Percentage of dyserythropoietic cells found in bone marrow aspirates on admission and at convalescence. n = 200 erythroblasts. ND = not detected. **D**. Representative images of dysplasic nuclei (upper left), a cytoplasmic bridge between erythroblasts (lower left), and erythroblasts presenting binucleated or budding nuclei (upper and lower right) (Giemsa-stained slides).
(TIF)

S2 Fig Bioinformatics pipeline and analyses tools. Read quality control was accessed by FastQC, and adaptor removal was performed using Cutadapt v1.4.2. Reads of length between 15 and 75 nucleotides were mapped to the human reference genome GRCh37.75 downloaded from the Ensembl database. HTSeq-count v0.6.0 was used to count and compare aligned reads to annotated human genes. Mapped reads were aligned to the precursor and mature datasets from MirBase v21.0 using Bowtie 2 v2.2.4. Samtools v0.1.18 and in-house Perl scripts were used to count aligned reads and to normalize gene expressions by library size.
(TIF)

## References

[1] Mueller I, Galinski MR, Baird JK, Carlton JM, Kochar DK, Alonso PL et al (2009) Key gaps in the knowledge of Plasmodium vivax, a neglected human malaria parasite. Lancet Infect Dis 9: 555–566. 10.1016/S1473-3099(09)70177-X 19695492

[2] Marchiafava E, Bignami A. (1894) On summer-autumnal fever The New Sydenham Society, London.

[3] Aitken GJ. (1943) Sternal pucture in the diagnosis of malaria. Lancet 2: 466–468.

[4] Wickramasinghe SN, Looareesuwan S, Nagachinta B, White NJ. (1989) Dyserythropoiesis and ineffective erythropoiesis in Plasmodium vivax malaria. Br J Haematol 72: 91–99. 2660903 10.1111/j.1365-2141.1989.tb07658.x

[5] Raina V, Sharma A, Gujral S, Kumar R. (1998) Plasmodium vivax causing pancytopenia after allogeneic blood stem cell transplantation in CML. Bone Marrow Transplant 22: 205–206. 10.1038/sj.bmt.1701299 9707032

[6] Salutari P, Sica S, Chiusolo P, Micciulli G, Plaisant P, Nacci A, et al (1996) Plasmodium vivax malaria after autologous bone marrow transplantation: an unusual complication. Bone Marrow Transplant 18: 805–806. 8899200

[7] O'Donnell J, Goldman JM, Wagner K, Ehinger G, Martin N, Leahy M, et al (1998) Donor-derived Plasmodium vivax infection following volunteer unrelated bone marrow transplantation. Bone Marrow Transplant 21: 313–314. 10.1038/sj.bmt.1701073 9489659

[8] Lacerda MV, Hipolito JR, Passos LN. (2008) Chronic Plasmodium vivax infection in a patient with splenomegaly and severe thrombocytopenia. Rev Soc Bras Med Trop 41: 522–523. 19009202 10.1590/s0037-86822008000500021

[9] Snounou G. (2002) Genotyping of Plasmodium spp. Nested PCR. Methods Mol Med 72: 103–116. 10.1385/1-59259-271-6:103 12125106

[10] Malleret B, Li A, Zhang R, Tan KS, Suwanarusk R, Claser C, et al (2015) Plasmodium vivax: restricted tropism and rapid remodeling of CD71-positive reticulocytes. Blood 125: 1314–1324. 10.1182/blood-2014-08-596015 25414440 PMC4401350

[11] Martin-Jaular L, Elizalde-Torrent A, Thomson-Luque R, Ferrer M, Segovia JC, Herreros-Aviles E, et al (2013) Reticulocyte-prone malaria parasites predominantly invade CD71hi immature cells: implications for the development of an in vitro culture for Plasmodium vivax. Malar J 12: 434 10.1186/1475-2875-12-434 24289105 PMC4220676

[12] Beurskens M, Mens P, Schallig H, Syafruddin D, Asih PB, Hermsen R, et al (2009) Quantitative determination of Plasmodium vivax gametocytes by real-time quantitative nucleic acid sequence-based amplification in clinical samples. Am J Trop Med Hyg 81: 366–369. 19635900

[13] Pava Z, Handayuni I, Wirjanata G, To S, Trianty L, et al (2015) Expression of Plasmodium vivax crt-o is related to parasite stage but not ex vivo chloroquine susceptibility. Antimicrob Agents Chemother 60:361–367. 10.1128/AAC.02207-15 26525783 PMC4704153

[14] Abdulsalam AH, Sabeeh N, Bain BJ. (2010) Immature Plasmodium falciparum gametocytes in bone marrow. Br J Haematol 85: 943. 10.1002/ajh.21796 20687103

[15] Aguilar R, Magallon-Tejada A, Achtman AH, Moraleda C, Joice R, Cistero P, et al (2014) Molecular evidence for the localization of Plasmodium falciparum immature gametocytes in bone marrow. Blood 123: 959–966. 10.1182/blood-2013-08-520767 24335496 PMC4067503

[16] Joice R, Nilsson SK, Montgomery J, Dankwa S, Egan E, Morahan B, et al (2014) Plasmodium falciparum transmission stages accumulate in the human bone marrow. Sci Transl Med 6: 244re245. 10.1126/scitranslmed.3008882 PMC4175394 25009232

[17] Hattangadi SM, Wong P, Zhang L, Flygare J, Lodish HF. (2011) From stem cell to red cell: regulation of erythropoiesis at multiple levels by multiple proteins, RNAs, and chromatin modifications. Blood 118: 6258–6268. 10.1182/blood-2011-07-356006 21998215 PMC3236116

[18] Lawrie CH. (2010) microRNA expression in erythropoiesis and erythroid disorders. Br J Haematol 150: 144–151. 10.1111/j.1365-2141.2009.07978.x 19912217

