# Supplementary material for: Plasmodium vivax gametocytes in the bone marrow of an acute malaria patient and changes in the erythroid miRNA profile
Source: PLoS Negl Trop Dis. 2017 Apr 6;11(4):e0005365. doi: 10.1371/journal.pntd.0005365 (PMC5383020; doi:10.1371/journal.pntd.0005365)
Supplement: S1 Table — (PDF) [file pntd.0005365.s001.pdf]

Table S1. Hematological and biochemical parameters from peripheral blood on admission and convalescence.

Parameter	D0	D42
Hematocrit (%)	41,32	46,87
Hemoglobin (g/dL)	13,98	15,86
Leukocytes (/mm3)	8300	9900
Platelets (/mm3)	74000	293000
Reticulocytes (%)	0,7	1,3
Creatinine (mg/dl)	1,3	0,9
Bilirubin (mg/dl)	1,67	0,5
LDH (IU/L)	975	340
Alkaline phosphatase (IU/L)	553	222
TGO (IU/L)	65	60
TGP (IU/L)	42	38
Urea (mg/dl	55	20
C-reactive protein (mg/L)	<6,5	<6,5
HIV	Negative	Negative
