# Supplementary figures and images for: Plasmodium vivax gametocytes in the bone marrow of an acute malaria patient and changes in the erythroid miRNA profile
Source: PLoS Negl Trop Dis. 2017 Apr 6;11(4):e0005365. doi: 10.1371/journal.pntd.0005365 (PMC5383020; doi:10.1371/journal.pntd.0005365)

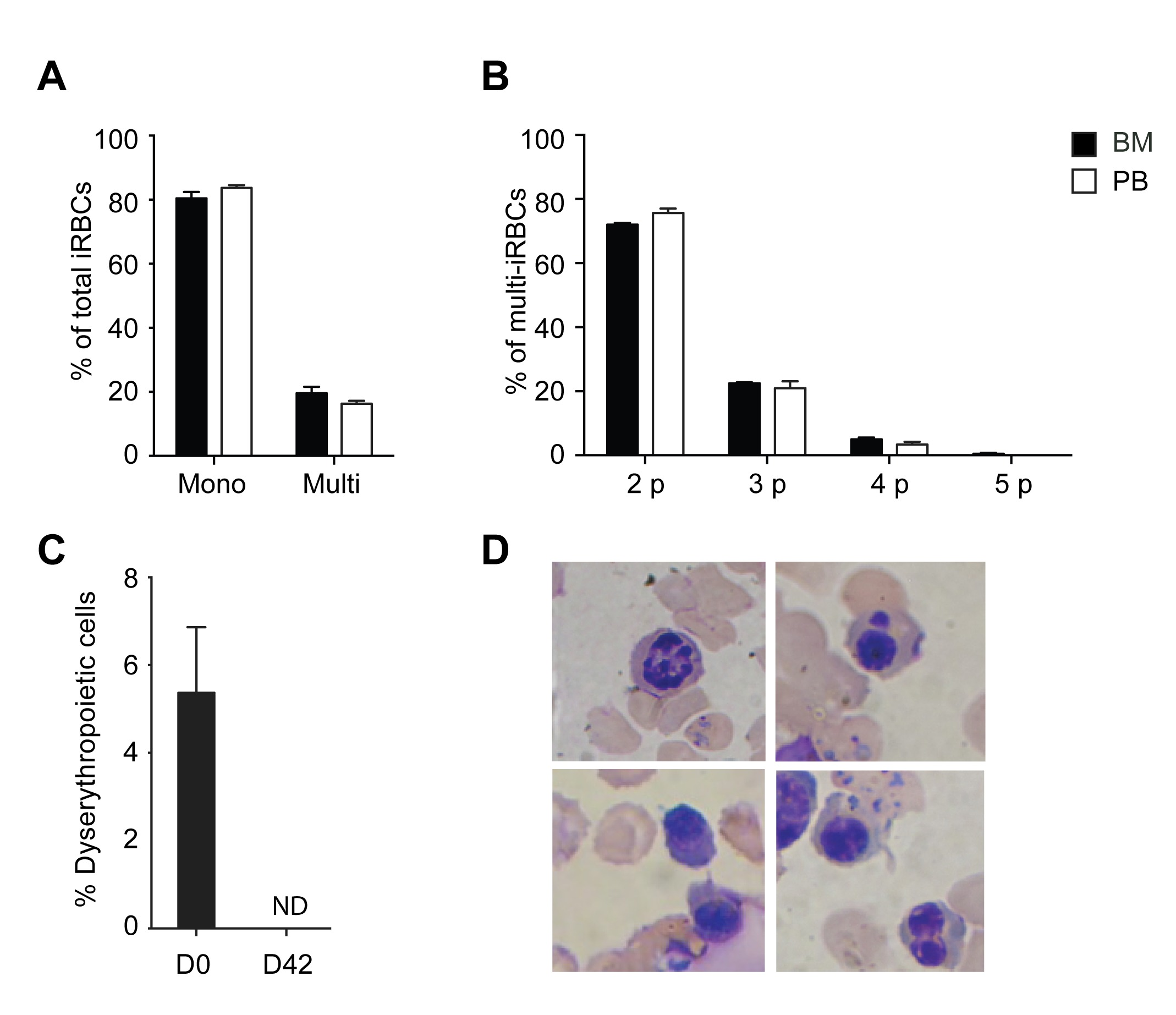

Supplement: S1 Fig — A. Percentages of single vs multiple ring-infected cells in the bone marrow and peripheral blood. B. Numbers of rings in individual infected cells in bone marrow and peripheral blood. Only infected cells containing multiple ring stages were used for confident counting (n = 500 iRBCs), although multi-invasion was observed in all parasite stages. C. Percentage of dyserythropoietic cells found in bone marrow aspirates on admission and at convalescence. n = 200 erythroblasts. ND = not detected. D. Representative images of dysplasic nuclei (upper left), a cytoplasmic bridge between erythroblasts (lower left), and erythroblasts presenting binucleated or budding nuclei (upper and lower right) (Giemsa-stained slides). (TIF) [file pntd.0005365.s002.tif]

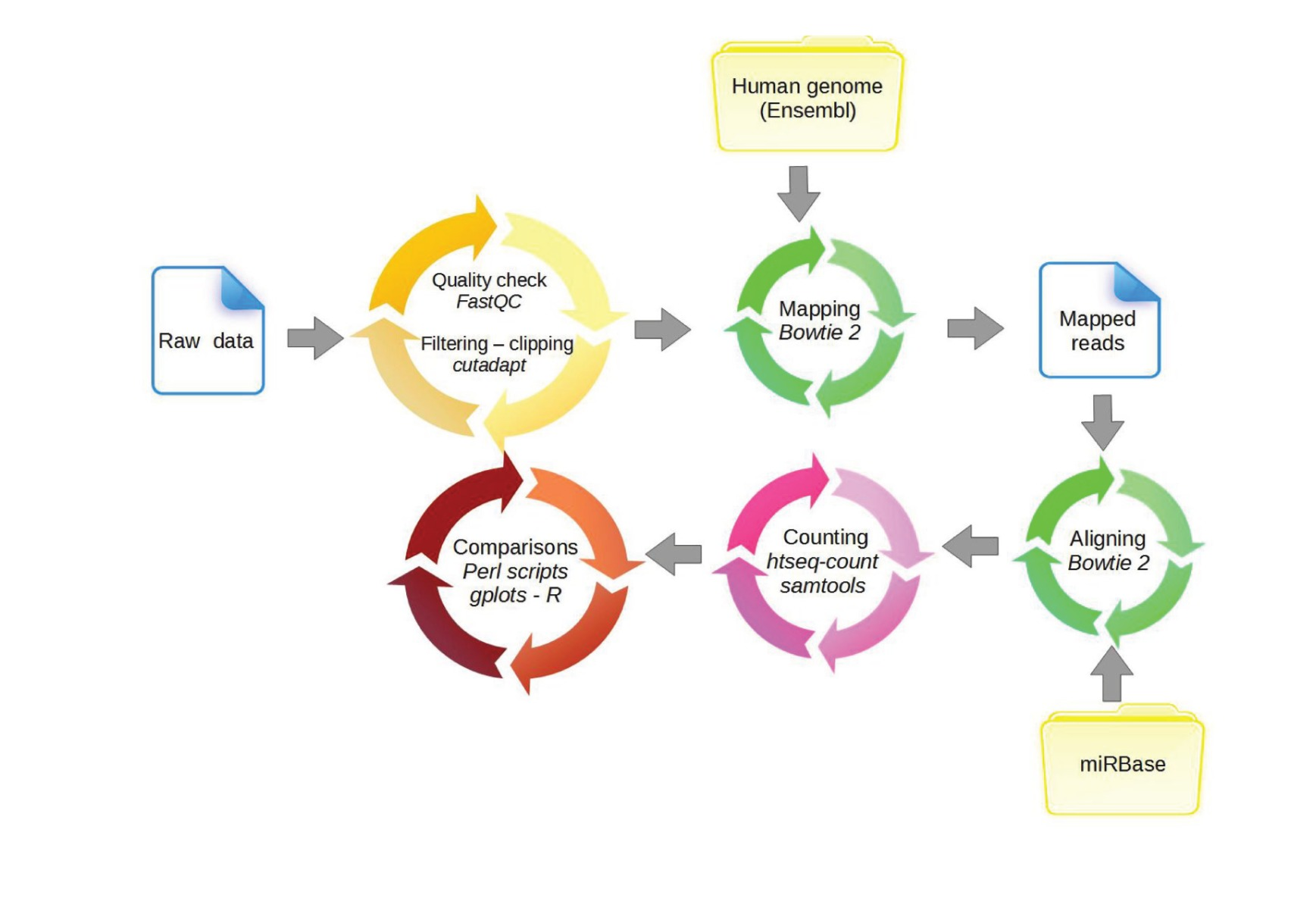

Supplement: S2 Fig — Read quality control was accessed by FastQC, and adaptor removal was performed using Cutadapt v1.4.2. Reads of length between 15 and 75 nucleotides were mapped to the human reference genome GRCh37.75 downloaded from the Ensembl database. HTSeq-count v0.6.0 was used to count and compare aligned reads to annotated human genes. Mapped reads were aligned to the precursor and mature datasets from MirBase v21.0 using Bowtie 2 v2.2.4. Samtools v0.1.18 and in-house Perl scripts were used to count aligned reads and to normalize gene expressions by library size. (TIF) [file pntd.0005365.s003.tif]
